# Considerations for Expanding Research and Clinical Care for the Neurodivergent Gender Diverse Population

**DOI:** 10.7759/cureus.51060

**Published:** 2023-12-25

**Authors:** Kashia A Rosenau, Erin Baroni, Elliot G Keenan, Maria L Massolo

**Affiliations:** 1 Internal Medicine/Pediatrics, University of California Los Angeles David Geffen School of Medicine, Los Angeles, USA; 2 Education, University of California Los Angeles, Los Angeles, USA; 3 Anthroplogy, Kaiser Permanente, Oakland, USA

**Keywords:** clinical care, gender diversity, neurodiversity, gender, autism

## Abstract

Little is currently known about the relationship between gender diversity and neurodiversity, although a growing body of researchers and clinicians are searching for more information to better serve this population. Gender-diverse individuals are three to six times more likely than cisgender individuals to identify as autistic or to report possible undiagnosed autism or autistic traits. Many gender-diverse individuals experience a shortage of gender-affirming medical care and are disproportionately impacted by barriers to mental health services. Similarly, autistic individuals report that the most common barrier to care is a lack of knowledgeable providers and/or resistance from providers to tailor care toward their specific needs. Two key areas in need of further research are 1) clinical approaches to gender-affirming medical and mental health care for neurodivergent patients and 2) the prevention and treatment of suicidality in gender-diverse neurodivergent individuals. Increasing collaborations amongst gender-diverse neurodivergent individuals, researchers, and clinicians are needed in order to further research and clinical practice to most directly and effectively improve physical and mental health care for the gender-diverse neurodivergent patient population.

## Editorial

Emerging research shows a unique relationship between gender diversity and neurodiversity, especially autism. Gender-diverse individuals are three to six times more likely than cisgender individuals to identify as autistic or to report possible undiagnosed autism or autistic traits [1]. Little is currently known about the relationship between gender diversity and neurodiversity, although a growing body of researchers and clinicians are searching for more information to better serve this population. Many gender-diverse individuals experience a shortage of gender-affirming medical care and are disproportionately impacted by barriers to mental health services [2]. Similarly, autistic individuals report the most common barrier to care is a lack of knowledgeable providers and/or resistance from providers to tailor care toward their specific needs [3]. It is imperative for clinical and research decision-making to include the voices of gender-diverse neurodivergent individuals. Further, the dearth of health care and mental health care providers who feel competent in providing care for these communities raises grave concerns about the quality of care being provided at the intersection of autism and gender diversity. Two key areas in need of further research are 1) clinical approaches to gender-affirming medical and mental health care for neurodivergent patients and 2) the prevention and treatment of suicidality in gender-diverse neurodivergent individuals.

While research, albeit limited, shows high co-occurrence rates between neurodiversity and gender diversity [4-5], the medical literature lacks research specifically on neurodivergent individuals seeking gender-affirming medical care. Although some providers elicit an informed consent-based and patient-centered approach when providing gender-affirming medical treatment(s), others still over-pathologize autism and gender diversity, while instituting restrictive measures that hinder access to gender-affirming care. Given the need for neurodiversity-oriented and gender-affirming care for gender-diverse neurodivergent patient populations, implementing a model of care with a focus on understanding an individual patient's needs and targeting care accordingly is more appropriate than focusing solely on strict and inflexible criteria that overgeneralize the needs of the patient population.

More research is needed in this area to further support an appropriate and inclusive model of care, as well as to further elucidate nuances that may need to be considered in neurodivergent and gender-diverse populations. One example could be a hesitance to receive hormone treatments by injection, given high rates of sensory distress and needle phobia [6-7]. There is a need for providers to work collaboratively with patients to find ways to best achieve medical goals and ensure barriers to care are reduced. Tools and resources have been created by the Academic Autistic Spectrum Partnership in Research and Education (AASPIRE) [8] and the Autistic Self-Advocacy Network (ASAN) [9], among others, that can enable patients to efficiently communicate accommodation preferences and patient needs to physical and mental health care providers.

A wealth of data shows that attempted and completed suicides are markedly more prevalent in gender-diverse youth than in cisgender youth [10]. Likewise, suicide rates are further elevated in gender-diverse autistic individuals [11]; according to recent research on suicidality in autistic youth, gender-diverse autistic females have the greatest risk of suicidality of any autistic group studied [12]. Research has found that suicide attempts by gender-diverse individuals are predicted by family rejection, with those considered to have experienced a high level of family rejection being three times more likely to have attempted suicide compared to other gender-diverse individuals [13]. For those that exist at the intersection of gender diversity and neurodiversity, there may be a tendency for family members and others to reject an individual’s gender-diverse identity, instead of affirming it, based on their neurodivergent status, with many families questioning whether gender is a current “fixed interest” [14].

The increased rates of suicidality in gender-diverse and neurodivergent patient populations warrant scientific inquiry into the prevalence, prevention, and treatment of suicidal thoughts and behaviors in gender-diverse neurodivergent individuals. A study on the first clinical program for neurodivergent gender-diverse adolescents was recently published by Strang et al. [15]. They utilized a Community-Based Participatory Research (CBPR) [16] approach to create specific considerations for practice and outline clinical approaches to facilitate support group sessions for parents and neurodivergent gender-diverse youth [15]. There is a great need for additional participatory research to further highlight the clinical needs of this population in order to decrease barriers to care and provide psychoeducation to families and support providers.

The current co-occurrence rates of gender diversity and neurodiversity highlight the need for additional research to better understand this relationship and to inform clinical practice. The Autism Intervention Research Network on Physical Health (AIR-P) is increasing initiatives to raise awareness, disseminate current information and educational resources, encourage additional research, and foster productive conversations surrounding the intersection of neurodiversity and gender diversity and the need for gender-affirming physical and mental health care from a neurodiversity lens [17]. Increasing collaborations amongst gender-diverse neurodivergent individuals, researchers, and clinicians are needed in order to further research and clinical practice to most directly and effectively improve physical and mental health care for the gender-diverse neurodivergent patient population.
